# Recent Progress in Dentistry

**Published:** 1886-07

**Authors:** William Herbert Rollins


AMERICAN JOURNAL

OF

DENTAL SCIENCE.

VOL. XX. THIRD SERIES.—JULY, 1886. No. 3.

ARTICLE I.

### RECENT PROGRESS IN DENTISTRY.

BY WILLIAM HERBERT ROLLINS.

*Danger of Pulpitis from the use of Gutta-percha as a Pulp Capping.*[1]

The writer claims that this material expands so much, that when used over a pulp, under a metal filling, it creates enough pressure to kill the pulp.

*Cystic Tumor of the Alveolar Membrane.*[2]

The patient had been treated by one physician for gout, by another, for an obscure liver disease, but in each case, without relief to the symptoms, which were intense pain over the face, scalp, and occiput, extending down the spine as far as the last ribs; and upon the neck, as far as the bifurcation of the two heads of the origin of the sterno-

1 Brit. Jour. of Dental Science, October, 1885.

2 Harris in Brit. Jour. of Dental Science, p. 894, 1885.

mastoid muscle. The pain was worse at night, and about half an hour after meals, at which latter time the stomach became tympanitic, impeding the movements of the diaphragm to such an extent as to cause alarming dyspnœa. An examination of the mouth showed nothing abnormal in its appearance. On a more extended examination, the resistance offered by a first upper molar to pressure, was found to be slightly different from that of the other teeth. On this slight hint, the tooth was extracted. Attached to the root, was found a hard, slightly vascular tumor with three lobes, which projected into the antrum. As the result of the extraction, all the symptoms disappeared.

*Loss of Sensation in the Gums and Lips after Tooth Extraction.*[3]

The patient, a surgeon, in the course of five years, had three teeth extracted, following which, in each case, there had been loss of sensation in the gums and lips, from the angle of the jaw forward to the median line. Sensation gradually returned without treatment, and was fully re-established in three months.

*Chronic Ptyalorrhœa of the Glands of the Oral Mucosa.*

This disease has not been clearly recognized, though to the effects which it produces upon the teeth several names have been given, beginning with John Hunter, who mistook one of its symptoms for a distinct disease to which he gave the name "decay by denudation." This name is still in use, and other and more recent writers have farther withdrawn attention from the real disease by giving the names "surface wear," "erosion," "denuding," "chemical abrasion," to its effects upon the teeth.

The only treatment which the writers who have used these names have suggested, has been either to do nothing, or, when the effects upon the teeth have been extensive to fill the cavities with gold.

---

3 Glassington in Dental Record, p. 193, 1886.

Ptyalorrhœa shows itself in an increase in the amount of the secretion of the acinous glands of the lips and cheeks. Accompanying this increase in amount is an increased viscidity and slight acidity. Even in those cases where the effect upon the teeth is very rapid there is seldom a marked acid reaction in the secretion. This faint acidity explains why the grooves in the teeth almost always are smooth, as if polished; indeed, many writers mistaking the real cause of the trouble, have attributed the grooving of the teeth to the effects of a stiff brush in cleaning them.

There are cases where caries supplements ptyalorrhœa in which the starting point of the caries is due to the intensity of the ptyalorrhœa, the evidently softened tooth substance not being removed as rapidly as formed; thus affording a culture ground for germs which produce the usual results, caries. These cases are chiefly those of the channeled form, in which the effects upon the teeth consist of grooves across the teeth near the necks.

Ptyalorrhœa is an entirely distinct disease from caries. In most cases it is a local expression of some ill-defined constitutional condition or conditions. In a less number of cases it is sharply local. Its effects upon the teeth show themselves in at least three forms. In a general wasting away of the teeth; in transverse channeling of the teeth at their necks; by the formation of saucer-shaped cavities upon the lbial surfaces of the teeth. This last form attacks the canine teeth often before any others. This fact, together with another, that pulpless teeth are as much affected as those having living pulps, tend to show that the effects upon the teeth are not due, as has been suggested, to a retrograde metamorphosis in which the wasting of the teeth is produced by an absorption of the lime salts by a new cellular growth. In one of these cases there is no living tissue from which the cells could come, while on the other hand it is not likely that if this were the true explanation the canine tooth which is the strongest tooth would be attacked first. In the cases in which the cupped or saucer shaped cavities are formed on prominent portions of the labial surfaces it is often easy to see the effect of the grandular congestion: the orifices on the mucous membrane occupying the centres of little raised papillæ of a deep red color. If the surface of the membrane is dried, only a few seconds will elapse before it is studded with little pearls of secretion, acid to litmus paper. In an ordinary condition of these glands a minute or more would elapse before any secretion would be observed, nor would the reaction ever be acid except early in the morning. It is an open question whether the action of the parotid secretion and also that of these glands may not be normally acid for a short time before rising; whether this transient acidity is due to the same acid or acids that are present in the pathological conditions named is not known. Treatment where the disease is simply a local manifestation of an undetermined constitutional condition, we can give alkalies as these at least diminish the local effects upon the teeth, though this treatment may be only palliative.

In those cases where the disease is evidently due to an overworked condition of the patient, as is frequently the case in nervous children, these alkalies are of great value, as they improve the condition of the digestion. A large number of cases of ptyalorrhœa of this kind have been cured in a few months by this treatment. In other cases where gout is responsible for the trouble this treatment is also of value.

*Local Treatment.* This should consist of the daily use of astringent and alkaline mouth washes. Where the disease is evidently local and confined to a few glands these may be partly broken up by tattooing, or the use of electrolysis may be suggested.

### *Fungi found in the Human Mouth.*

Miller[4] has found twenty-two fungi in the human mouth. Ten of these are micro- or diplo-cocci. Five are

---

4 In Ind. Pract., p. 283, 1885.

bacteria, and six are bacilli. In liquid media three develop into leptotrix, one into spirilli; eight are motile, while only three have been seen to form spores; the others multiplying only by division. Ten are strictly ærobian, and eight grow equally well without air. Sixteen produce an acid reaction in beef extract and in sugar; four produce alkaline reaction. In all cases where the acid produced was tested it was found to be lactic acid. His conclusions are, that a great majority of the fungi found in the human mouth are capable of producing acid from cane or grape sugar but in non-fermentable substances the reaction produced is alkaline In those cases where the fungus produces both an acid and an alkaline reaction there are two processes going on, first the nutrition of the organism accompanied with an alkaline reaction; second, the fermentive action accompanied by acid products. A large number of these fungi possess a peptonizing action which accounts for the solution of the dentine after it has been decalcified.

Any of these fungi that can produce acid by fermentation of carbo-hydrates, or can dissolve the decalcified dentine may aid in the production of caries, while only those which combine both of these properties, can produce the disease. A solution of dentine or enamel without previous decalcification cannot take place. The comparative or complete independence of air of many of these germs explains why caries can go on under fillings or deep in the cavity of a tooth. The fact that teeth form such poor culture substrata for schizomycetes is an additional proof of the position that they are not the primary cause of caries which is produced by acid resulting from the action of these organisms on certain carbo hydrates in the mouth.

D

*Nitrous Oxide Dangerous.*[5]

Nitrous oxide should never be given pure, as, though it may not produce death, there are many concomitant ac-

---

5 Lafont in South. Dent. Jour. page 75, 1886.

cidents. Among those mentioned are, first, death of the fœtus in a young woman enciente five months, who had taken the gas to have a tooth extracted. Second, several cases of albuminuria. Third, numerous cases of suppression of the menses.

*Alkalies for Sensitiveness of the Teeth.*[6]

If bicarbonate of potash is given in doses of five grains three times a day the sensibility of the teeth in excavating them is much diminished.

*Iodol.*

Among the new antiseptics, this drug has been useful in treating dead pulps and as an injection in Rigg's disease.

*Napelline in Acute Pulpitis.*[7]

Napelline was given in granules of one-twelfth of a grain, repeated every fifteen minutes. In each of the cases the pain ceased after five or six dozes had been given.

*Naphthol.*

B. naphthol is superior to carbolic acid or creosote as an injection into the sac of an alveolar abscess, and as a dressing for suppurating pulps. Its comparative freedom from odor and the absence of unpleasant after-effects if it comes in contact with the gums or lips are strong points in its favor.

*Amalgam Fillings.*[8]

Fletcher says mercurial poisoning may occur where a plug is made with the grossest carelessness and an immense amount of uncombined mercury, though he has never seen a case in twenty years practice. But this, even if it does occur is a proof not that amalgam is in fault but that the dentist is not acquainted with the material he is using. Any dentist who will put in a filling saturated with uncom-

---

6 Abbot in Dental Pract. page 54, 1886.

7 Grognot in South. Dent. Journ., p. 388, 1885.

8 In Am Journ. Dent. Science.

bined mercury had better discontinue practice till his education is more complete.

*Ambrosia Artemesæfolia as a Hæmostatic.*[9]

Several cases are given in which a decoction of the fresh leaves used locally and taken internally stopped the bleeding when other remedies had failed. No exact directions are given.

*Pilocarpin in Toothache.*[10]

A solution of two grains of the drug in one ounce of water was used. This was injected into the temporal region on the side of the neuralgia from the teeth. In one case a quarter of a grain was used, in two others one-eighth of a grain.

In all the cases the pain disappeared in about an hour after the injection.

*Arsenical Treatment of the Tooth Pulp.*[11]

The usual length of time that arsenic has been allowed to remain in contact with the tooth has been a few hours only. Then, if as usually happened, the pulp was not dead, the application was renewed as many times as was necessary to produce this result. Morsman thinks the arsenic should remain in contact with the tooth until the pulp is killed even if it takes a month. He claims there is no danger in this, as the arsenic is never absorbed.

---

9 Hill in Ohio State Journ. of Dent. Science, p. 479, 1885.

10 Kuszakoff in Dent. Register page 150, 1886.

11 Morsman in Am. Journ. of Dental Science page 453, 1886.

